# Assessment of the utility of underwater hyperspectral imaging for surveying and monitoring coral reef ecosystems

**DOI:** 10.1038/s41598-023-48263-6

**Published:** 2023-11-30

**Authors:** Matthew S. Mills, Mischa Ungermann, Guy Rigot, Joost den Haan, Javier X. Leon, Tom Schils

**Affiliations:** 1 Marine Laboratory, University of Guam, Mangilao, GU USA; 2 School of Science, Technology, and Engineering, University of the Sunshine Coast, Sippy Downs, QLD Australia; 3 PlanBlue GmbH, Bremen, Germany; 4 Hamburg, Germany

**Keywords:** Marine biology, Coral reefs, Tropical ecology, Imaging, Imaging and sensing

## Abstract

Technological innovations that improve the speed, scale, reproducibility, and accuracy of monitoring surveys will allow for a better understanding of the global decline in tropical reef health. The DiveRay, a diver-operated hyperspectral imager, and a complementary machine learning pipeline to automate the analysis of hyperspectral imagery were developed for this purpose. To evaluate the use of a hyperspectral imager underwater, the automated classification of benthic taxa in reef communities was tested. Eight reefs in Guam were surveyed and two approaches for benthic classification were employed: high taxonomic resolution categories and broad benthic categories. The results from the DiveRay surveys were validated against data from concurrently conducted photoquadrat surveys to determine their accuracy and utility as a proxy for reef surveys. The high taxonomic resolution classifications did not reliably predict benthic communities when compared to those obtained by standard photoquadrat analysis. At the level of broad benthic categories, however, the hyperspectral results were comparable to those of the photoquadrat analysis. This was particularly true when estimating scleractinian coral cover, which was accurately predicted for six out of the eight sites. The annotation libraries generated for this study were insufficient to train the model to fully account for the high biodiversity on Guam’s reefs. As such, prediction accuracy is expected to improve with additional surveying and image annotation. This study is the first to directly compare the results from underwater hyperspectral scanning with those from traditional photoquadrat survey techniques across multiple sites with two levels of identification resolution and different degrees of certainty. Our findings show that dependent on a well-annotated library, underwater hyperspectral imaging can be used to quickly, repeatedly, and accurately monitor and map dynamic benthic communities on tropical reefs using broad benthic categories.

## Introduction

Shallow-water tropical reefs harbor the highest biodiversity of marine ecosystems on the planet^1,2^ and are characterized by high species richness, high levels of endemism^3^, and considerable functional group variability^4^. Local and global stressors have caused a continued decline in reef health over recent years^5–11^. Amidst this global trend of declining reef health and rapid environmental change, the need for fast, efficient, and accurate assessments of reef communities is more pressing than ever before^12^. Many methods to optimize reef community monitoring have been developed and implemented over the years, each with their own tradeoffs, advantages, and disadvantages^13–17^. Reef monitoring has historically been conducted through repetitive identifications and annotations of benthic categories by investigators. One of the more common methods is the phototransect methodology, which is still widely used and often regarded as the baseline standard for the evaluation of new techniques^18,19^. Although photoquadrat surveys typically cover small expanses of reef, they require extensive post-collection processing and analysis. Moreover, it is difficult to investigate several characteristics that could drive reef community dynamics by using photoquadrats alone, such as species/taxa distributions, habitat patchiness and clustering, or environmental context.

Globally, in-situ reef surveys are estimated to have only been carried out in approximately 0.01% to 0.1% of the world’s coral reef regions, and much of the data comprise observations at a resolution of one to ten meters^20^. However, the emergence of new technologies has enabled the development of a suite of alternative approaches to benthic monitoring^16,21^. The flexibility of remote sensing has led to a wealth of alternative methods using a variety of instruments including satellites^14^, aircrafts^22^, drones^23^, submersibles^24^, and handheld sensors^25^. Some approaches included the use of active remote sensing via radar^26^ or LiDAR^27^, while others employed passive remote sensing using RGB or multispectral imagery^14–16,28,29^, and hyperspectral imaging^30–35^. These studies generated massive spatial and temporal datasets^36^ that required novel analysis methods. Machine learning is one such method that has been used to effectively analyze these large datasets^37^. Machine learning has been employed in multiple ecological studies and has produced positive results for species identification, predictive modelling, biomonitoring, and habitat mapping^34,35,38–44^. It has also allowed researchers to process hyperspectral data in reef monitoring studies^25,45^.

A diver-operated hyperspectral imager, the HyperDiver, was developed by the Max Planck Institute (Bremen, Germany) with the aim of leveraging hyperspectral imaging and machine learning to automatically map benthic communities^25^. This initial study served as a proof-of-concept for the use of this methodology. Though conducted on biodiverse reefs in Papua New Guinea, the habitat map was generated for a single transect that primarily consisted of scleractinian corals and a few other functional groups^25^. Subsequent studies have produced promising results suggesting that this methodology could serve as a viable method for fast and reliable reef monitoring and mapping^46,47^. However, many of these surveys were conducted on Caribbean reefs with much lower taxonomic diversity compared to the biodiverse reefs in the tropical Pacific.

To increase the scope and utility of underwater hyperspectral imaging, PlanBlue GmbH (Bremen, Germany) started to develop the DiveRay as of 2017. The DiveRay was used to conduct reef surveys in Guam (13° 28ʹ N, 144° 46ʹ E), the largest and southernmost island of the Mariana Archipelago. Positioned just outside the Coral Triangle^48^, Guam is home to more than 5000 documented marine species^3,49–52^, making it one of the most species-rich ecosystems of all US jurisdictions. Much like other reef systems globally, the health of Guam’s reefs have seen a steady decline since the 1960s^53^. However, this decline has accelerated since 2013, resulting in an estimated 34–37% decline in total coral cover^54–56^ and substantial changes in reef composition^57^. Surveys were conducted on eight reefs in Guam with the aim of evaluating the utility of a diver-operated hyperspectral imager and machine learning as a proxy for benthic reef monitoring. This was done by comparing the hyperspectral results to traditional photoquadrat surveys, focusing primarily on accuracy across two levels of benthic category identification. The advantages and considerations of both survey methodologies for reef mapping and monitoring were evaluated and discussed.

## Materials and methods

### Study area

**Figure 1:**
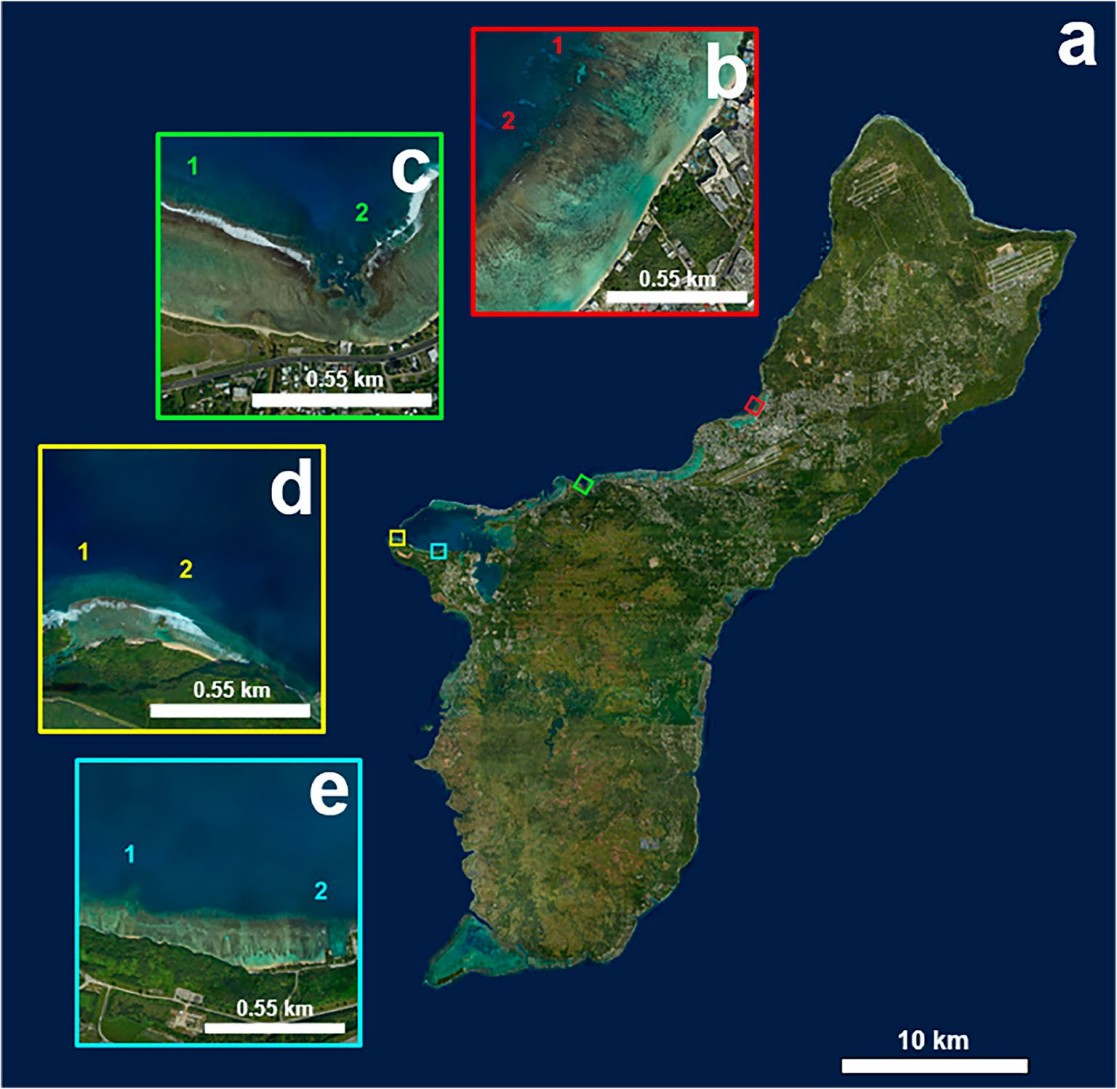
Map of Guam. (**a**) Map of Guam indicating the study sites. Paired sites are identified by colored squares. Sites numbered 1 indicate fish spawning aggregation sites (FSAS), and sites numbered 2 indicate non-fish spawning aggregation sites (NFSAS). (**b**) Sites in Tumon Bay (red square). (**c**) Sites at Asan (green square). (**d**) Sites at Orote Point (yellow square). (**e**) Finger Reef (1) and Gab Gab Reef, (2) in Apra Harbor (blue square). Maps were created using ArcGIS Pro version 2.9 (Esri, Redlands, CA; www.esri.com).

Benthic surveys were conducted in October 2019 at eight fringing reefs (8–12 m in depth) along the west coast of Guam (Fig. 1). The selected sites covered a range of ecological and environmental factors, including human impact, habitat composition and heterogeneity, and wave exposure. The sites were originally surveyed as part of a project examining the differences between reefs where fish spawning aggregations were known to occur (FSAS; Fig. 1b–e, sites numbered ‘1’) and adjacent reefs where spawning aggregations have not been observed (NFSAS; Fig. 1b–e, sites numbered ‘2’). However, since those differences are not being examined herein, the survey sites simply represent a spatially diverse set of reef areas for the purpose of this study. As such, the distinction of FSAS and NFSAS only serves to differentiate between survey sites. Two types of benthic survey methods, photoquadrat and hyperspectral imagery, were performed at each site. The photoquadrat surveys were conducted to ground-truth the results of the hyperspectral surveys by comparing the model-predicted communities of the hyperspectral surveys to those derived from the photoquadrat surveys. The hyperspectral and photoquadrat surveys were conducted concurrently, which allowed for a direct comparison between both methods.

### Photoquadrat survey method

At each site, six 50 m transects were established and surveyed using the phototransect method. The start and end points of each transect were georeferenced manually. The full length of each transect was georeferenced using the DiveRay (see below). Photos of 0.25 m^2^ quadrats were taken at every meter along a transect, cropped, and post-processed to improve contrast and color. Each of the photos were overlaid with 20 nonaligned, systematically sampled points, yielding a total of 300 photos and 6000 sample points per site. 128 benthic categories were used to identify (1) organisms to the highest taxonomic resolution possible (typically genus- or species-level) and (2) abiotic reef substrates (Supplementary Table S1).

The 128 categories were aggregated into 10 taxonomic or functional groups: Scleractinia, Corallinophycidae, Peyssonneliales, other red macroalgae, green macroalgae, brown macroalgae, Porifera, octocorals/hydrozoans, other benthic invertebrates, and turf algae/cyanobacteria/bare substrate. These groups were used to compare the two survey methods using broad benthic categories that could be used in rapid macroscale reef monitoring or ecological assessments, comparable to either “Level 1” or “Level 2” community data as outlined by the Global Coral Reef Monitoring Network (GCRMN)^58,59^. The same categories were also aggregated into 52 groups consisting primarily of genera, species, species complexes, and abiotic reef substrates. This was done to group related organisms that are difficult to distinguish from one another without in-depth morphological or genetic analysis (e.g., massive *Porites* species, *Dipsastraea* spp.), taxa that are difficult to identify in photoquadrat images (e.g., Porifera species), or taxa for which many species are undescribed (e.g., red algae belonging to the Corallinophycidae and Peyssonneliales). These groups, considered “Level 3” categories by the GCRMN, were used to compare the reef communities at a high taxonomic resolution^58,59^.

### Hyperspectral survey method

**Figure 2:**
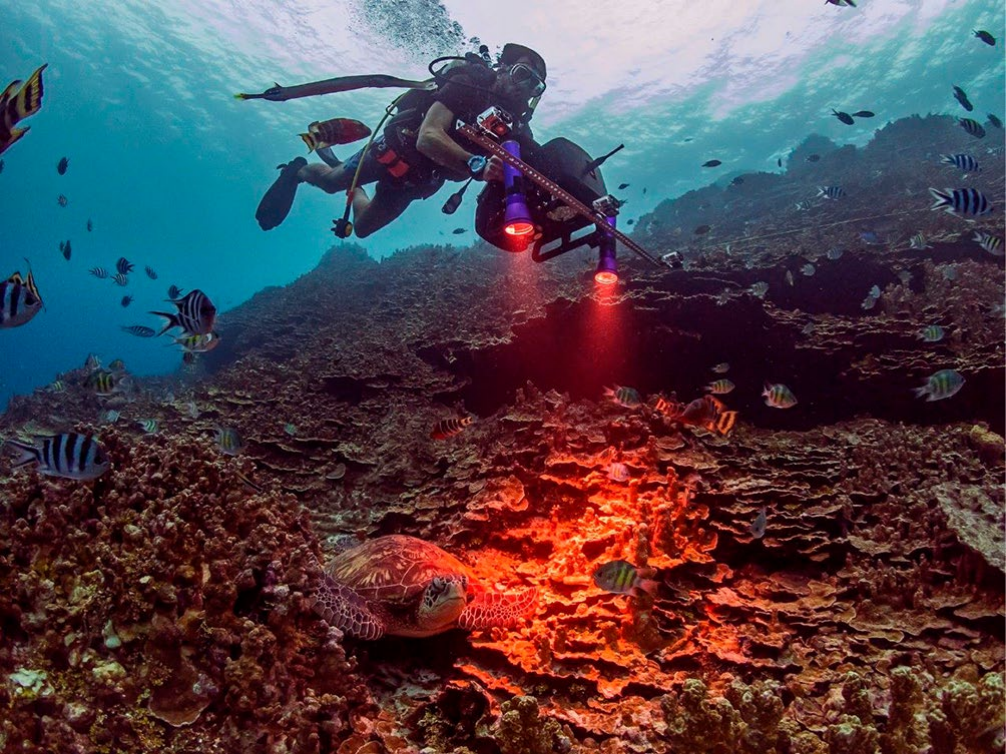
DiveRay survey at Finger Reef FSAS (Fig. 1e, site number 1).

A beta version of an underwater hyperspectral imager, the DiveRay, was used to scan transects at each of the eight study sites (Fig. 2; PlanBlue GmbH, Bremen, Germany). Informed consent was obtained from the researcher pictured in Fig. 2 for the publication of this image in an online open access publication. The DiveRay is a diver-operated hyperspectral camera that combines four primary components when recording hyperspectral data: (1) a push-broom or line-hyperspectral camera, meaning that each frame records a line orthogonal to the swimming direction with a field of view of roughly 30 degrees and a spectral resolution of approximately 2.9 nm (680 spatial pixels); (2) external full-spectrum lights (Keldan GmbH, Brügg, Switzerland) that allow for the collection of hyperspectral data independent of lighting conditions; (3) a 5 MP low noise CMOS reference (RGB-)camera that records at a frame rate of 30 Hz to assist in the annotation of hyperspectral data; and (4) a combination of inertial, acoustic, and magnetic navigation sensors, fused by an algorithm developed by PlanBlue, that allow data to be georeferenced with a relative accuracy of within ± 0.5 m while scanning. With a swath area of 50 m long and approximately one meter wide, the DiveRay was used to scan 300 m^2^ of reef at each survey site. The resulting scans are large (upwards of 4–6 gigabytes per scan), as each recorded pixel contains complete spectral information about the visible and near-infrared part of the spectrum.

In a first processing step, recorded spectra were smoothed and standardized. Wavelengths without sufficient signal were removed (i.e., primarily the longest wavelengths since most of the signal gets absorbed by water). Spectra were then normalized to account for incident light using the recorded signal of a 10 × 10 cm matte grey reference plate that was placed in the survey area of each transect. Assuming incident light does not change significantly during the survey, this normalization of spectra allows for a comparison between transects at a site. To account for depth, surveys followed the topography of the reef, where the DiveRay was oriented approximately one meter above, and parallel to, the varying reef terrain at any given time. This was done primarily to reduce the inaccuracies and errors resulting from large variations in reef height because the quality and range of spectral data are influenced by the optical environment and the height from which hyperspectral images are captured^25^.

User annotation effort was required before machine learning algorithms could be used to automatically classify the benthic communities. Hyperspectral scans were visualized in the HyperSuite software package (PlanBlue GmbH), which also served as the platform to annotate hyperspectral images and discern benthic categories. 91 benthic categories were recognized in the hyperspectral analysis, as some benthic categories were visually indistinguishable from one another in the hyperspectral scans (e.g., branching *Acropora* species). However, these categories were consistent between the photoquadrat and hyperspectral survey methods, as they were used to compare the predicted benthic communities across two distinct degrees of taxonomic resolution (Supplementary Table S1). Nearly 3550 regions of interest (ROIs) were delineated, each identifying one of the 91 benthic categories. These ROIs covered an approximate total of 1.02 million pixels across all hyperspectral scans and were used as the library to train machine learning algorithms prior to automated classification. ROIs were grouped into the same taxonomic categories as described above. A deep-learning based spectral-spatial Residual Neural Network (ResNet) model, provided by PlanBlue, was used for supervised classification^60^. The architecture and pre-activation concepts of this ResNet are based on Zhong et al.^61^ and He et al.^62^, respectively. The model used the information from multiple spectra in a patch around a central pixel to create spectral and model-determined spatial features to identify categories. This was done by initially reducing channel dimension via 1 × 1 convolutions. Spectral features were then created via residual units^63^ along the last dimension. The number of spectral features were then adjusted before spatial features were created via residual units on the adjusted features. The final classifier was created through average pooling over the window and softmax layer. 90% of the labeled pixels were used to train the model to identify benthic categories, while the remaining 10% of labeled data were used as a validation set to assess how well the model performed on non-training data. The trained model was then tested on the rest of the scans to predict a label for every recorded pixel in each transect, resulting in estimates of benthic community composition for each transect.

Each benthic category prediction per pixel is accompanied by a certainty value. These values indicate the model’s confidence in the correct category identification of a pixel. In other words, the higher the certainty value, the less likely that spectral signatures of other benthic categories match that of the pixel in question. These values can be used to determine a threshold for pixel selection. The scale went from no threshold value (identifications for all pixels irrespective of their certainty value) to the highest threshold of 99% (i.e., pixels of which the model is at least 99% certain that it could not be assigned to another category). The benthic cover estimates derived from the hyperspectral scans were then compared to those of the photoquadrat surveys to evaluate using the DiveRay as an alternative to photoquadrat surveys.

### Data analyses

All statistical analyses were done using R version 4.1.3 (R Core Team, Vienna, Austria) and RStudio version 2022.02.1 + 461 (RStudio Team, Boston, MA, USA). Analysis of similarities (ANOSIM) tests were performed using the ‘vegan’ package to test whether there were statistical differences between the communities estimated using the two surveys at each site. This was done by using a ranked dissimilarity matrix with defined groups (survey method) to compare the mean rank within groups (transects) to the mean rank between groups^64^. By ranking dissimilarities, ANOSIM compliments non-metric multidimensional scaling (nMDS) approaches to evaluating community data^64^. For the communities identified using broad benthic groups, the significance of differences in the estimated cover of Scleractinia between survey methods were tested using a one-way ANOVA and the Tukey’s honest significant difference (Tukey’s HSD) post hoc test. Differences in community assemblages between survey methods were visualized using nMDS plots based on Bray–Curtis dissimilarity matrices and overlaid with hulls around each site. Broad group community differences were plotted without transformation in order to best compare survey methods. However, communities described using high resolution categories were typically dominated by few taxa and contained a high number of categories with (very) low cover. To reduce the large discrepancies in cover between groups, high resolution groups were visualized using square root transformed cover data.

## Results

### Characterization of reef communities

Scleractinian corals represented the highest benthic cover at three sites: Finger Reef FSAS (54 ± 5%), Gab Gab NFSAS (55 ± 5%), and Orote NFSAS (42 ± 6%) but covered much less reef at the other five sites, ranging from 8 ± 4% (Tumon FSAS) to 25 ± 11% (Tumon NFSAS). While coral communities were species rich (over 50 identified taxa separated into 26 high resolution ID categories), they were largely dominated by a combination of massive *Porites* species, *Porites rus*/*monticulosa*, and *Leptoria phrygia*. Five sites were dominated by pavement/turf algae/cyanobacteria, which made up between 59 ± 9% (Tumon NFSAS) and 80 ± 6% (Orote FSAS) of the substrate cover on these reefs. These two categories accounted for over 78% of the total benthic cover across all sites, while the presence of other abundant organisms (≥ 5% average cover) varied between sites. One site, Orote NFSAS, possessed a high abundance of green macroalgae, which was mostly comprised of *Halimeda* spp. (20 ± 7% cover). Sponges were present in high abundance at Finger Reef FSAS (9 ± 3%), Gab Gab NFSAS (8 ± 2%), and Orote FSAS (5 ± 2%), but the species composition differed between these sites. The sponge communities present on Finger Reef FSAS and Gab Gab NFSAS were primarily comprised of *Melophlus sarasinorum*, *Rhabdastrella globostellata*, and *Xestospongia carbonaria*, while other Porifera species (e.g., *Cliona* and *Dysidea* spp.) occurred in high abundance at Orote FSAS. Lastly, members of the reef-building and cementing Corallinophycidae were present in relatively high abundance at Asan NFSAS (8 ± 2%), Tumon FSAS (6 ± 2%), and Tumon NFSAS (6 ± 2%). No other substrate categories occurred in abundances greater than 2.5% at the surveyed reefs.

### Optimal certainty

**Figure 3:**
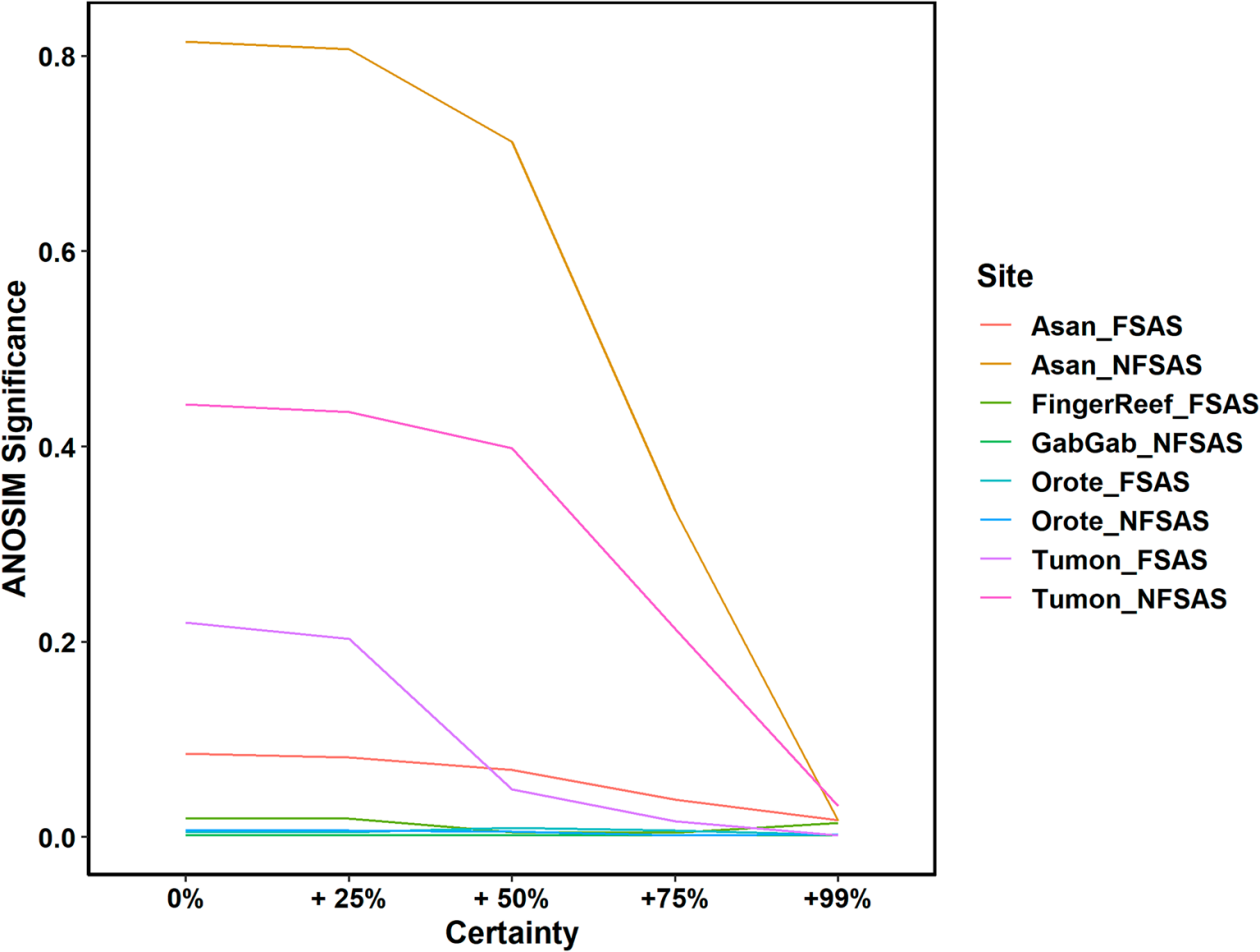
Line graph plotting the ANOSIM significance (y-axis) comparing photoquadrat and DiveRay community estimates for each survey site across varying certainty values (x-axis).

Prior to running further analyses, the optimal certainty threshold needed to be determined. This was done by examining the ANOSIM results of the comparison between the photoquadrat-derived community metrics and those predicted from the hyperspectral scans for a series of certainty value thresholds (intervals of 5%, from no threshold to > 99%; Fig. 3). For all sites, little change was observed from communities predicted without certainty threshold to those with 25% threshold certainty. At certainty values above 25%, a significant drop in similarity (ANOSIM significance) was observed for most sites, suggesting that limiting the dataset to certainty thresholds higher than 25% was not suitable for further analyses. In fact, for most sites, the similarity between survey methods was highest when no threshold was placed on certainty (Fig. 3). As such, the model-predicted categories without thresholds placed on certainty were used for all subsequent analyses.

### Broad group category predictions

**Figure 4:**
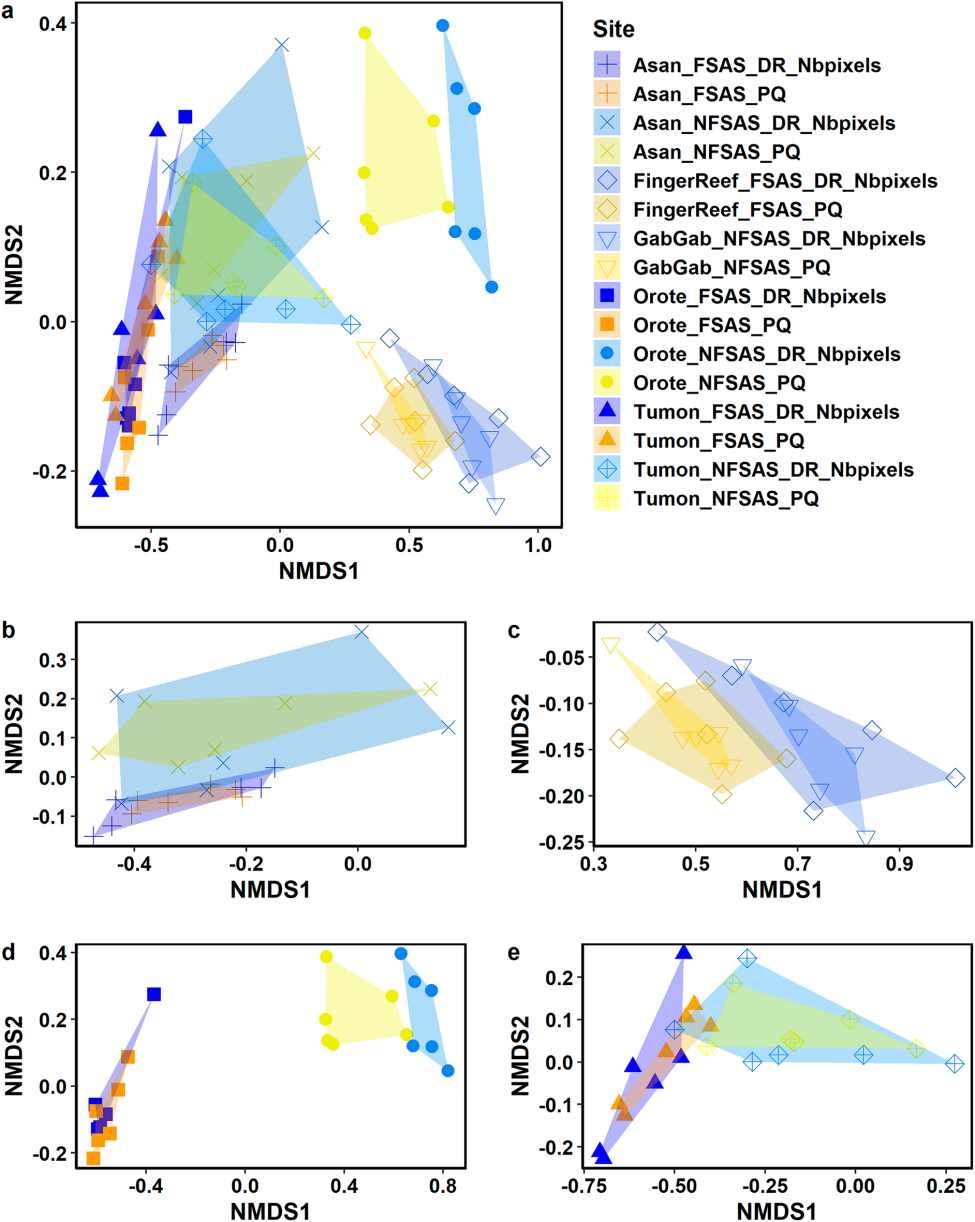
nMDS plots of cover data of broad benthic categories with hulls overlaid for each of the survey methods and sites. DiveRay surveys are denoted by “DR”, and photoquadrat surveys are denoted using “PQ”. Plots display transects at (**a**) all sites, (**b**) Asan, (**c**) Apra Harbor, (**d**) Orote Point, and (**e**) Tumon Bay. Transects surveyed using hyperspectral imaging are shaded blue and those surveyed by photoquadrats are colored in yellow-orange.

For two survey sites, Finger Reef FSAS and Gab Gab NFSAS, the community estimates differed significantly between survey methods across all analyses. These two sites are unique when compared to all other survey sites, highlighting considerations for the use of the DiveRay in certain habitats. The community analyses for these two sites were only included when based on identifications for broad benthic groups (Fig. 4; Supplementary Table S2). The differences between the two survey methodologies were even greater for the more detailed categories.

**Figure 5:**
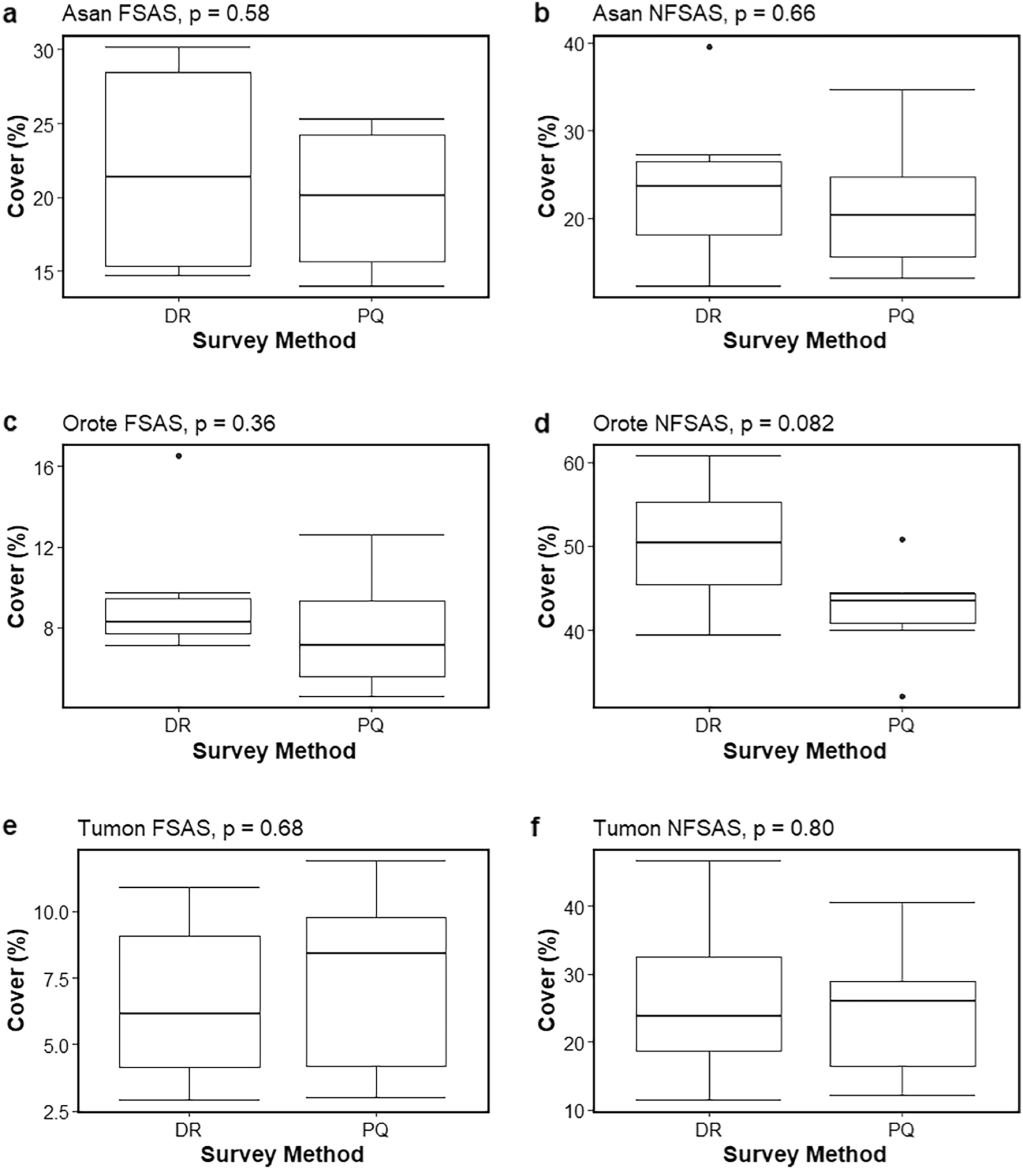
Box plots of scleractinian coral cover identified in the photoquadrat (PQ) and DiveRay (DR) surveys for each site. Site names and the p-values of one-way ANOVA tests are listed on top of each plot. Boxes show upper and lower quartiles, horizontal lines show the median values, whiskers represent the range excluding outliers, and dots denote outliers.

Apart from Finger Reef FSAS and Gab Gab NFSAS, the predictions of the broad benthic categories based on the hyperspectral data aligned well with those of the photoquadrat surveys (Fig. 4). Benthic cover estimates based on the automated classification of hyperspectral scans were similar to those derived from photoquadrat surveys for four sites: Asan FSAS (ANOSIM statistic R = 0.233, significance = 0.085), Asan NFSAS (ANOSIM statistic R = − 0.091, significance = 0.816), Tumon FSAS (ANOSIM statistic R = 0.076, significance = 0.214), and Tumon NFSAS (ANOSIM statistic R =  − 0.002, significance = 0.445). This was further improved when only comparing the predicted cover of scleractinian corals, where the model-predicted cover was found to not significantly differ from the photoquadrat-derived estimates at all six sites (Fig. 5). Model-predicted cover of Corallinophycidae, octocorals/hydrozoans, and other benthic invertebrates performed just as well and did not significantly differ across all six sites (Supplementary Table S4). Predicted cover of pavement/turf algae/cyanobacteria and red macroalgae were also largely similar between survey methods, where estimates did not differ significantly across five of the six sites (Supplementary Table S4). Cover predictions for the other four broad benthic categories differed significantly between methods across at least three of the six sites (Supplementary Table S4).

### High resolution category predictions

**Figure 6:**
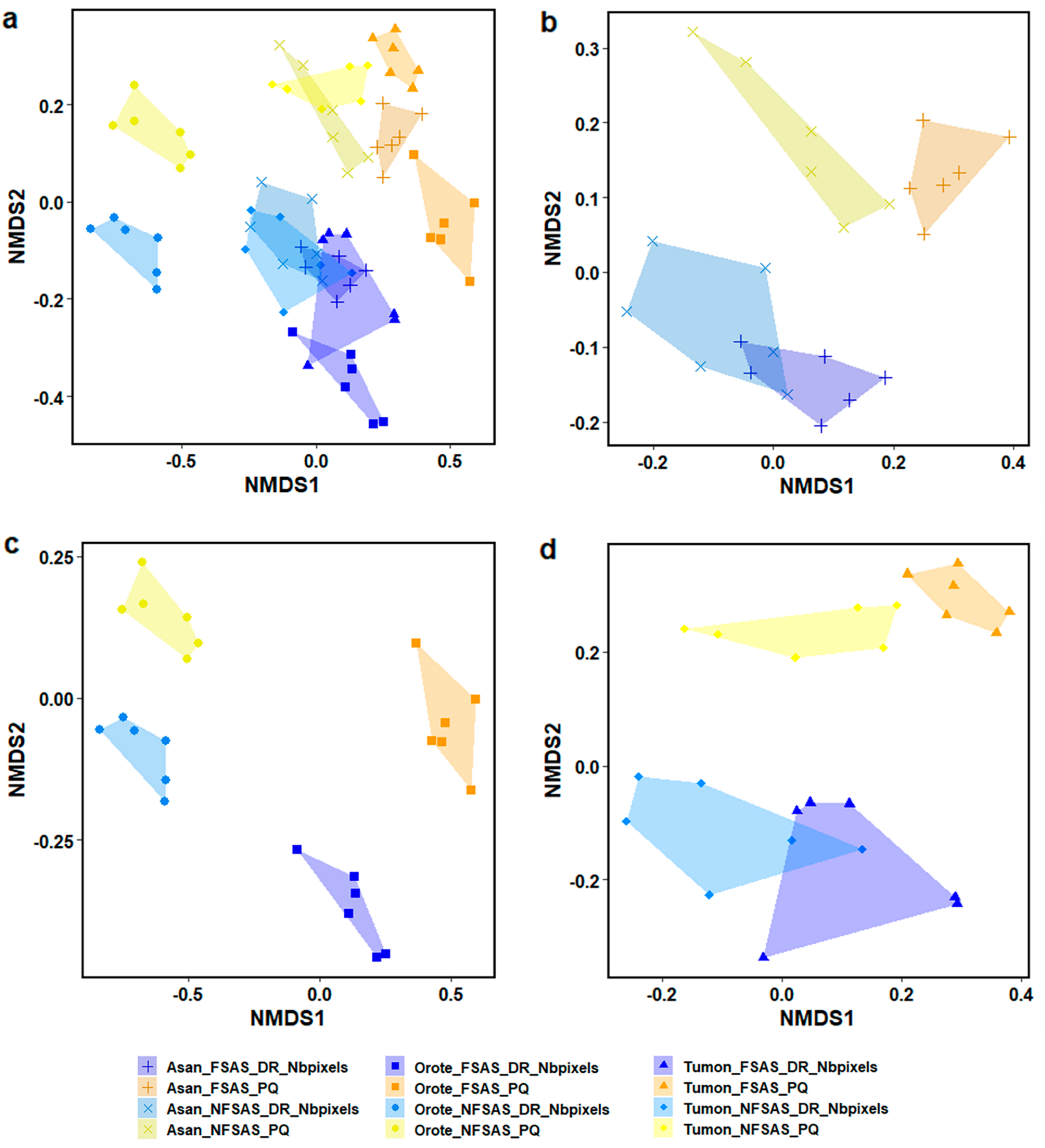
nMDS plots of square root transformed cover data of high resolution categories with hulls overlaid for each of the surveys. DiveRay surveys are denoted by “DR”, and photoquadrat surveys are denoted using “PQ”. Plots display transects at (**a**) all sites, (**b**) Asan, (**c**) Orote Point, and (**d**) Tumon Bay. Transects surveyed using hyperspectral imaging are shaded blue and those surveyed by photoquadrats are colored in yellow-orange.

Identifications at the highest taxonomic resolution in hyperspectral scans were less accurate (Fig. 6; Supplementary Table S3). The model-predicted community estimates did not significantly differ from those of the photoquadrat surveys for only two sites: Asan NFSAS (ANOSIM statistic R = 0, significance = 0.373) and Tumon NFSAS (ANOSIM statistic R = 0.167, significance = 0.095). Like the broad benthic category estimates, the hyperspectral results performed better when only examining scleractinian corals. Scleractinian coral communities estimated by photoquadrat and hyperspectral surveys were not significantly different for four sites: Asan NFSAS (ANOSIM statistic R = − 0.033, significance = 0.504), Orote NFSAS (ANOSIM statistic R = 0.146, significance = 0.117), Tumon FSAS (ANOSIM statistic R = 0.165, significance = 0.089), and Tumon NFSAS (ANOSIM statistic R = 0.007, significance = 0.362).

## Discussion

### Optimal certainty threshold

The purpose of this study was to evaluate the performance of automated image classification on hyperspectral images to optimize benthic reef surveys. The implementation of underwater hyperspectral imaging and machine learning algorithms for benthic reef monitoring is still a novel approach^25,35,47,65,66^. This study is the first to directly compare results from hyperspectral data with those generated from photoquadrat surveys across multiple sites at different levels of identification, while considering a range of certainty thresholds. The optimal certainty threshold of model-predicted data was determined before downstream analyses were performed. As certainty thresholds increased, the number of included pixels decreased rapidly (Supplementary Fig. S1). This would continue until, at the highest thresholds, only pixels of the most dominant and commonly occurring taxa (e.g., scleractinian corals, *Porites rus*/*monticulosa*, massive *Porites*, turf algae/cyanobacteria) were retained. Lower certainty thresholds retained less abundant taxa, which was apparent when comparing the community composition between sites (Fig. 3). The inclusion of less abundant taxa benefitted the similarity between the hyperspectral and photoquadrat surveys (Fig. 3).

### Reflection on the performance of a beta version of the DiveRay

The community estimates showed significant differences across all analyses for two survey sites: Finger Reef FSAS and Gab Gab NFSAS. This emphasizes the need to consider initial site selection carefully when using the DiveRay system. These two sites, located within the sheltered Apra Harbor (Fig. 1e), are unique when compared to the other six sites with regards to their benthic composition, habitat complexity, and environmental conditions^67,68^. Somewhat paradoxically, the benthic communities present on these reefs are more homogeneous, dominated by largely monospecific scleractinian coral communities comprised of *Porites rus*. The colonies of *P. rus* at these sites are phenotypically plastic, possessing three distinct color morphs, and exhibit high degrees of structural complexity. Moreover, with the exception of large sponges (*Melophlus sarasinorum*, *Rhabdastrella globostellata*, and *Xestospongia carbonaria*), many of the other benthic organisms present at these sites occurred in small heterogenous patches that were often situated between columns or plates of *P. rus*. This made identifying the small heterogenous patches of turf algae/cyanobacteria and other benthic organisms on the hyperspectral scans difficult at these sites. In addition, contrary to the other sites, these reefs also occur on a relatively steep slope, adding a significant vertical component to the reef structure. The reef pictured in Fig. 2 is characteristic of these two sites. Even with two external full-spectrum lights attached to the DiveRay, the structural complexity and slope at these two sites created a significant amount of shading on the hyperspectral scans. This further increased the difficulty of identifying benthic organisms in the hyperspectral scans. Shading was a minor issue for the photoquadrat method, which could explain why scleractinian coral cover was overestimated in the hyperspectral scans by the machine learning algorithms. These two sites highlighted structural complexity limitations for hyperspectral imaging and were excluded from further analyses.

Broad group community estimates differed significantly between survey methods for two other sites: Orote FSAS and Orote NFSAS. The reef community at Orote NFSAS was unique due to the high abundance of green macroalgae (Supplementary Table S2), the predicted cover of which was consistent between both survey techniques. Apart from that, the reef community was similar to those of Finger Reef and Gab Gab. The high abundance of *P. rus* and small heterogenous patches of turf algae/cyanobacteria at Orote NFSAS likely contributed to the difference in community estimates between the two methods. Orote FSAS was characterized by low coral and high turf algae/cyanobacteria cover (Supplementary Table S2). Sponges (Porifera), one of the categories whose predicted cover differed most between surveys, were also present in high abundances on the reef. The sponges inhabiting the reef (e.g., *Cliona* and *Dysidea* spp.) were small and difficult to identify on hyperspectral scans, which likely contributed to their underestimation in the model-predicted communities.

DiveRay data from 2019 did not sufficiently characterize benthic composition using high-resolution categories. The communities of only two sites (Asan NFSAS and Tumon NFSAS) were found to not differ significantly between both survey methods. The significant differences observed at the other four sites cannot solely be attributed to the machine learning model. A model can only be as effective as the input data from the annotation library. Limitations to delineate multiple ROIs in the hyperspectral scans for all high resolution categories might have been insufficient to account for the high biodiversity of Guam’s reefs. A similar study from Curaçao examined a subset of 31 hyperspectral transects from a dataset containing 147 hyperspectral transects from 8 nearshore reefs^46,47^. Though they tended to overestimate the cover of corals, turf algae, and sponges, the machine learning methods used in the study by Schürholz and Chennu^47^ were able to produce detailed habitat maps with reasonably high consistency. However, despite examining a comparable dataset spanning the same number of survey sites, the dataset from Guam had nearly twice the number of unique labels of benthic categories (91) compared to the 47 identified in the Curaçao study^46,47^. The significantly higher marine biodiversity in Guam suggests that a much more robust annotation library might be needed, which could be accomplished through increased sampling and annotation efforts.

While there were 91 benthic categories identified in the annotation libraries, the proportion of ROI identifications were unintentionally skewed toward the most dominant taxa. The annotation imbalance was so pronounced that the three most recorded ID categories (massive *Porites* spp., *Porites rus*/*monticulosa*, and pavement/turf algae/cyanobacteria) contained more pixels (~ 650,000 pixels) than the other 49 ID categories combined (~ 450,000 pixels). This imbalance in ROIs was also observed in the hyperspectral dataset of the Curaçao study, where the authors concluded that this could be avoided by prioritizing biodiversity coverage rather than spatial coverage across all transects^46^. The identification of ROIs used in our study was directed toward prioritizing both spatial and biodiversity coverage, suggesting that this imbalance could be better explained by other factors such as rarity of occurrence (e.g., *Pocillopora damicornis* and *Favites* spp.), small size (e.g., *Padina* spp.), or difficulty of identification on hyperspectral scans despite a pixel resolution of less than one centimeter (e.g., Peyssonneliales and *Lobophora* spp.). Consequently, the model-predicted identification of less abundant and smaller benthic categories was inconsistent and, somewhat paradoxically, tended to overpredict the occurrence of rarer categories. This could be due there not being enough pixels (ROIs) available to sufficiently train the model to distinguish these taxa from others, causing the model to confuse them with other more dominant categories. The identification accuracy of these categories is expected to improve with increased annotation effort, more surveying, or targeted scanning of less common taxa. Predictions based on hyperspectral data performed better when just comparing scleractinian coral communities using the high-resolution categories, where the communities at four sites (Asan NFSAS, Orote NFSAS, Tumon FSAS, and Tumon NFSAS) were found not to differ significantly. This is similar to results reported by Schürholz and Chennu^47^ and indicate that this methodology is effective in differentiating corals at a high taxonomic resolution. The coral communities at these four sites were dominated by massive *Porites* species, *Porites rus*/*monticulosa*, and *Leptoria phrygia*. These three categories are abundant on Guam’s reefs, easily identifiable, and were well-represented in the annotation library. On the other hand, the coral communities of the other two sites (Asan FSAS and Orote FSAS) were more species rich, with 18 and 20 of the 26 scleractinian categories being found at each site respectively, while no more than 16 coral categories were recorded at the other four sites. It is also worth noting that the area of reef surveyed using the DiveRay was more than quadruple the area surveyed using photoquadrats, which could account for some differences in cover estimates.

Photoquadrat and hyperspectral surveys require similar field expertise. For non-repetitive small-scale survey campaigns, photoquadrat surveys are favored as they can provide a comprehensive baseline and the equipment is readily available. The same is true for collecting high taxonomic resolution benthic community data. These photoquadrat surveys have long been used for marine benthic studies^18,19,69^, but have their own disadvantages and limitations. Photoquadrat surveys are time-consuming and labor intensive. In this study, surveying six transects using the photoquadrat methodology took up to one hour to complete. In contrast, covering one dive site using the DiveRay took approximately 20 min. Marking, deploying, and retrieving transect lines is necessary for repeat surveys using photoquadrat or hyperspectral imaging. For non-repetitive surveys of large reef areas, however, the navigation sensors of the DiveRay eliminate the need for transect lines as they accurately record survey tracks that can readily be imported and mapped in geographic information systems. Photoquadrat surveys are also not ideal when surveying large areas or observing taxon-specific dynamics on reefs^70,71^, as the photos taken require a significant amount of post-processing and identification time (1–3 weeks per site in this study). Moreover, the photoquadrat methodology has been shown to underestimate the percentage cover of substrate categories^13,72,73^, which could also explain some of the discrepancies between the traditional survey method and the DiveRay surveys. Lastly, the surveys conducted for this study used a beta version of the DiveRay, which has undergone continuous hardware and software development since 2019. Studies have reported improved results as sensor technology improves^27,34,47^, and updates to the DiveRay and the HyperSuite software would likely improve classification success.

### Advantages of underwater hyperspectral imaging

Hyperspectral surveys of benthic reef communities could improve the speed of data collection and processing. It is often necessary to survey vast reef areas to properly assess biodiversity and spatial metrics such as patchiness and clustering^74,75^. Employing the traditional photoquadrat method for such expansive surveys would demand considerable time, effort, logistic, and financial resources. Though underwater hyperspectral surveys require less time in the field than photoquadrat surveys, building annotation libraries demands for an initial investment of time and effort (typically less than one week per site in this study). However, once established, the availability of annotation libraries is beneficial for longer term monitoring efforts as additional imagery can be analyzed within minutes to hours through the automated data processing pipeline. This is considerably faster than the 1–3 weeks needed to analyze the photoquadrats of one site. In addition, once an annotation library is built, the analysis of hyperspectral scans can be executed immediately after data collection. So, while the initial expenditure to conduct hyperspectral surveys seems higher than those of photoquadrat surveys, cost savings are to be expected for repetitive monitoring efforts that cover large areas^14,25^.

Results from hyperspectral surveys in Curaçao suggest that annotation libraries can be exchanged between survey efforts of similar reef environments^47^. As such, a more robust library of ROIs that better account for rare taxa and the high biodiversity in Guam could significantly improve model prediction accuracy, and thus drastically improve overall time-to-data in subsequent surveys. Scans can also be re-analyzed as annotation libraries are updated over time, which could further increase the prediction accuracy and temporal comparison with previously conducted surveys. Further, libraries built using survey data from a broad range of habitats over a large geographical area could standardize and reduce observer bias in monitoring efforts with a regional or even global focus.

The use of machine learning for automated annotation of benthic surveys has been shown to be effective when discerning broad or well-defined benthic categories^37,47^. The results of this study suggest the same, as benthic cover estimates based on hyperspectral surveys were most reliable when using broad benthic categories such as those often used by reef managers or in large-scale rapid ecological assessments^47,76^. The average community similarity ([1 − Bray–Curtis dissimilarity] × 100) between the different broad category estimates was high for all sites (Table 1), ranging from 75% (Gab Gab NFSAS) to 96% (Asan NFSAS). These results, as well as those reported by Schürholz and Chennu^47^, suggest that underwater hyperspectral imaging can accurately estimate benthic community composition at the broad category level, making it a useful method for rapid ecological assessments or large monitoring programs. The technique would be particularly useful to monitor live coral cover, which is one of the main indicators for the global decline in reef health^76–80^. Such rapid and drastic changes in reef composition emphasize the need to improve the quantity and frequency of reef monitoring efforts at local, regional, and global scales^59,81–83^. The fact that scleractinian coral cover predicted through hyperspectral imaging was similar to that estimated by photoquadrats for six of the survey sites (Fig. 5) suggests that the DiveRay is an effective tool to monitor, map, and predict coral cover. In addition to scleractinian corals, the hyperspectral and photoquadrat methods were largely congruent when predicting the cover of pavement/turf algae/cyanobacteria, Corallinophycidae, red macroalgae, octocorals/hydrozoans, and other benthic invertebrates. These categories, which were either abundant or easily distinguishable on hyperspectral scans, further reinforce the notion that underwater hyperspectral imaging could effectively and accurately survey reef communities using broad benthic categories. These results confirm the findings of Bryant et al.^84^, who concluded that automated and semi-automated technologies could be used to detect changes in reef condition like overall declines in coral cover. Further, while indicators of reef health (e.g., bleaching, disease, mortality) were not addressed in this study, hyperspectral imaging and machine learning algorithms can be used to track and quantify temporal changes in reef health after storms (or other damaging disturbance events), shifts in biodiversity (e.g., after invasive species outbreaks), or coral bleaching^85,86^. Given the often time-sensitive nature of coral reef surveys, the rapid data turnover enabled by underwater hyperspectral imaging can make this a valuable tool in future monitoring efforts.

**Table 1:** Similarity of estimated benthic communities derived from hyperspectral and photoquadrat surveys using broad benthic categories.

Study site	Similarity (%)
Asan FSAS	94.82
Asan NFSAS	96.08
Finger Reef FSAS	78.45
Gab Gab NFSAS	75.43
Orote FSAS	94.37
Orote NFSAS	76.8
Tumon FSAS	93.09
Tumon NFSAS	93.49

Similarity, calculated by subtracting Bray–Curtis dissimilarity from one then multiplying by 100, is listed for each study site.

**Figure 7:**
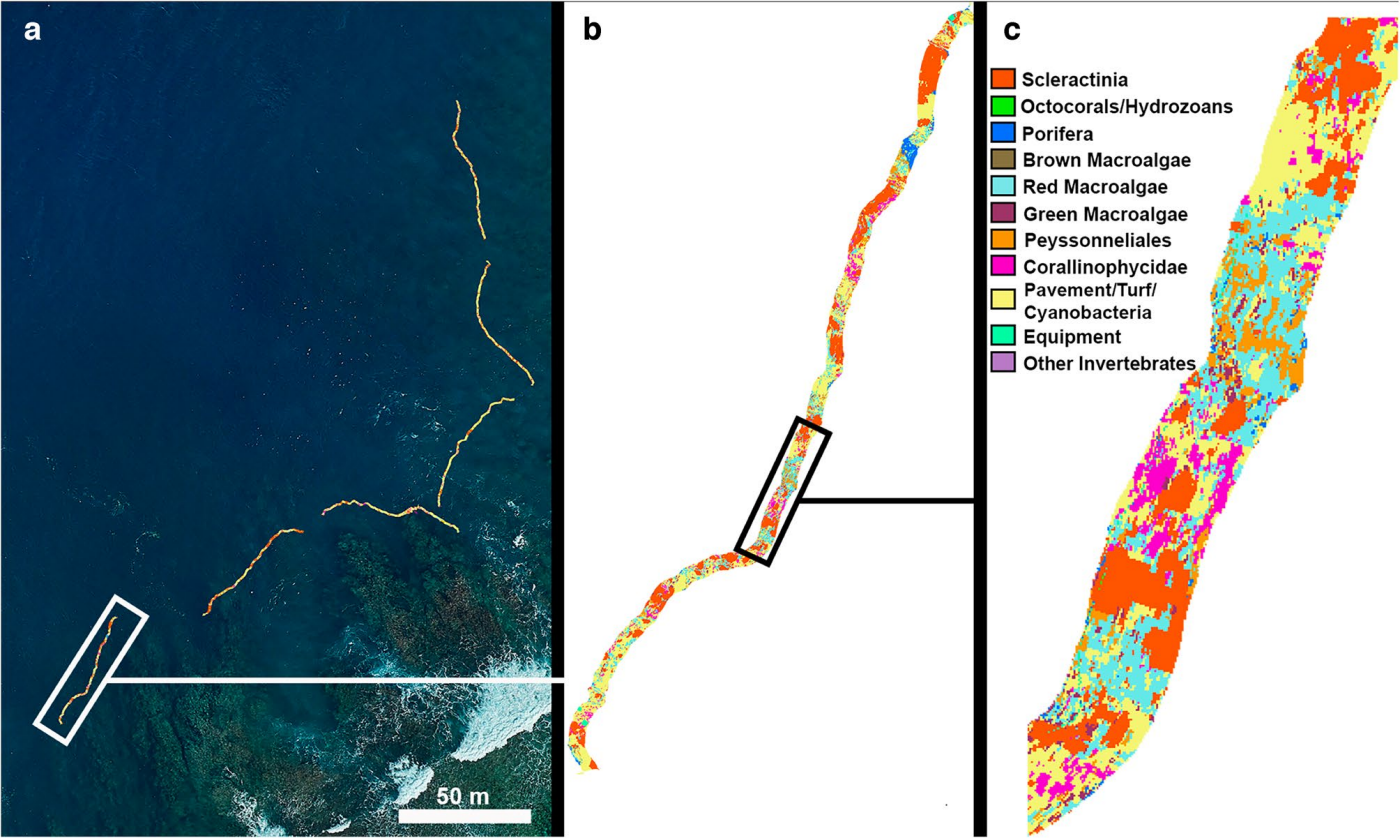
(**a**) Georeferenced habitat maps of transects surveyed using hyperspectral imaging at Asan NFSAS. (**b**) Close-up of one fully classified habitat map. Transect length = 50 m. (**c**) Further magnification of a portion of the benthic habitat map depicted in (**b**). Maps were created using ArcGIS Pro version 2.9 (Esri, Redlands, CA; www.esri.com).

The maps generated for hyperspectral scans also provide additional spatial data that are valuable for biodiversity, biomass, health, and habitat assessments^25,35^ (Fig. 7). These maps can be used to measure parameters relevant to the spatial ecology of phase shifts^87^, habitat degradation^88^, and habitat complexity and heterogeneity^89^. Other metrics derived from these habitat maps, like patchiness, clustering, or other spatial distribution parameters are increasingly important to better understand reef resilience^54,90^. Georeferenced maps also benefit the accuracy of repeat surveys and are valuable for ground-truthing satellite, aerial, and hydrographic products.

As is the case with other survey techniques^13,72^, there are both advantages and considerations to using close-range hyperspectral imaging to survey benthic reef communities. Based on the technology available in 2019, cover estimates derived from hyperspectral scans could not reliably predict benthic composition using high resolution categories (genus or species level). However, this capability is likely to improve as hardware technology advances (particularly sensor resolution and accuracy) and more extensive annotation libraries become available^47^. Hyperspectral imaging was already effective when predicting community composition using broad categories, particularly in estimating scleractinian coral cover. A strength of underwater hyperspectral imaging is its ability to generate detailed habitat maps at higher resolutions than afforded by above water techniques. So, when mindful of the type of habitat investigated and pending the availability of well-documented annotation libraries, underwater hyperspectral imagery is a promising tool for rapid, frequent, and accurate monitoring of reef communities.

### Supplementary Information

Supplementary Figure S1.

Supplementary Table S1.

Supplementary Table S2.

Supplementary Table S3.

Supplementary Table S4.

## Data Availability

Synthesized data used in downstream analyses can be found within the Supplementary Information files. However, due to substantial file and dataset sizes, the full (unsynthesized) datasets generated and/or analyzed during this study are available from the corresponding author on reasonable request.
