# Supplementary material for: Assessment of the utility of underwater hyperspectral imaging for surveying and monitoring coral reef ecosystems
Source: Sci Rep. 2023 Nov 30;13:21103. doi: 10.1038/s41598-023-48263-6 (PMC10689744; doi:10.1038/s41598-023-48263-6)
Supplement: Supplementary file 1 — Supplementary Figure S1. [file 41598_2023_48263_MOESM1_ESM.docx]

**
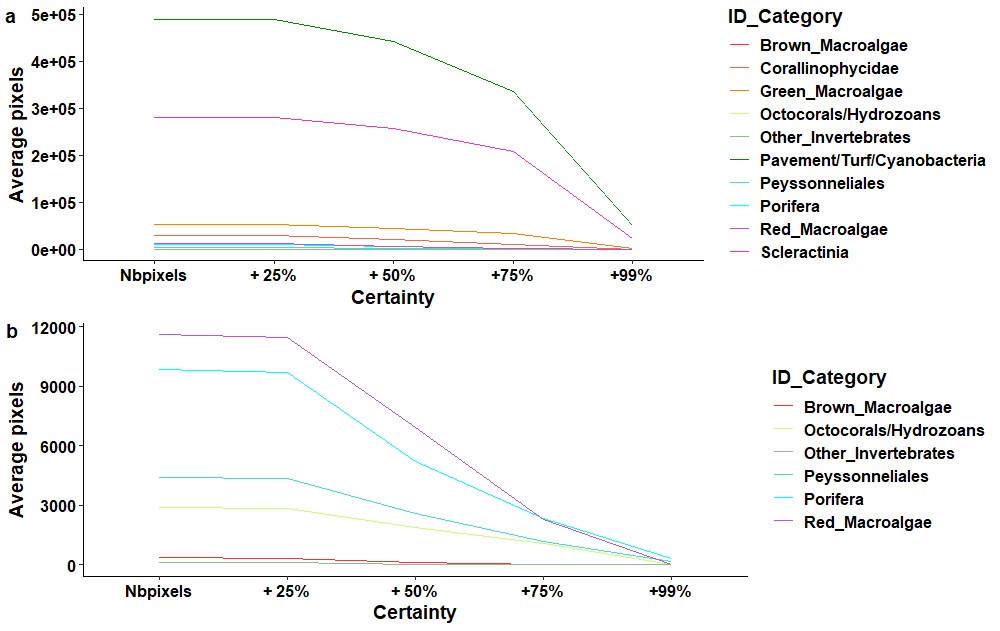
**

**Supplementary Figure S1** Line graphs plotting the average number of identified pixels per broad ID category (y-axis) as certainty level increases (x-axis). (a) Plot including all broad ID categories showing rapid decrease in pixels as certainty increased. (b) Plot with the four largest ID categories (Scleractinia, Pavement/turf/cyanobacteria, Corallinophycidae, Green macroalgae) removed showing the significant decrease or complete loss of less dominant taxa.
